# Vimentin affects inflammation and neutrophil recruitment in airway epithelium during *Streptococcus suis* serotype 2 infection

**DOI:** 10.1186/s13567-023-01135-3

**Published:** 2023-01-30

**Authors:** Yu Meng, Shaojie Lin, Kai Niu, Zhe Ma, Huixing Lin, Hongjie Fan

**Affiliations:** 1 MOE Joint International Research Laboratory of Animal Health and Food Safety, College of Veterinary Medicine, Nanjing Agricultural University, Nanjing, China; 2 Jiangsu Co-Innovation Center for Prevention and Control of Important Animal Infectious Diseases and Zoonoses, Yangzhou University, Yangzhou, China

**Keywords:** *Streptococcus suis* serotype 2, vimentin, airway epithelium, inflammation, neutrophils, NF-κB signaling

## Abstract

*Streptococcus suis* serotype 2 (SS2) frequently colonizes the swine upper respiratory tract and can cause Streptococcal disease in swine with clinical manifestations of pneumonia, meningitis, and septicemia. Previously, we have shown that vimentin, a kind of intermediate filament protein, is involved in the penetration of SS2 through the tracheal epithelial barrier. The initiation of invasive disease is closely related to SS2-induced excessive local inflammation; however, the role of vimentin in airway epithelial inflammation remains unclear. Here, we show that vimentin deficient mice exhibit attenuated lung injury, diminished production of proinflammatory cytokines interleukin-6 (IL-6), tumor necrosis factor-alpha (TNF-α), and the IL-8 homolog, keratinocyte-derived chemokine (KC), and substantially reduced neutrophils in the lungs following intranasal infection with SS2. We also found that swine tracheal epithelial cells (STEC) without vimentin show decreased transcription of *IL-6*, *TNF-*α, and *IL-8*. SS2 infection caused reassembly of vimentin in STEC, and pharmacological disruption of vimentin filaments prevented the transcription of those proinflammatory cytokines. Furthermore, deficiency of vimentin failed to increase the transcription of nucleotide oligomerization domain protein 2 (NOD2), which is known to interact with vimentin, and the phosphorylation of NF-κB protein p65. This study provides insights into how vimentin promotes excessive airway inflammation, thereby exacerbating airway injury and SS2-induced systemic infection.

**Supplementary Information:**

The online version contains supplementary material available at 10.1186/s13567-023-01135-3.

## Introduction

*Streptococcus suis* (*S. suis*), a Gram-positive bacterium, is a major swine pathogen that causes septicemia, meningitis, and pneumonia, responsible for severe global financial losses in the swine industry [1, 2]. *S. suis* is also an emerging zoonotic agent [1]. It causes streptococcal toxic shock-like syndrome (STSLS) in humans who have close contact with diseased pigs or consume contaminated raw pork products [3]. In Southeast Asian countries, such as Thailand and Vietnam, *S. suis* has become the leading cause of human meningitis [3, 4]. Based on bacterial surface capsular polysaccharide antigens, *S. suis* is divided into 29 serotypes [5, 6]. Among these identified serotypes, *S. suis* serotype 2 (SS2) has a high isolation rate among clinically infected pig and human cases and is considered one of the most virulent serotypes [7]. SS2 usually asymptomatically colonizes the upper respiratory tract of swine and can cause systemic inflammation through the airway epithelium [3].

The airway epithelium is an important sentinel and effector of innate immunity [8]. Detection of invading pathogens or multiple stimuli results in the liberation of proinflammatory and chemotactic cytokines by airway epithelium contributing to leukocyte invasion [9, 10]. Activation of signaling cascades in airway epithelial cells leads to the production of cytokines and chemokines such as IL-6, CXCL5, CXCL8 (IL-8), and CXCL10 [11–13]. These cytokines and chemokines facilitate the recruitment of dendritic cells, macrophages, and neutrophils to the site of infection to eliminate invading pathogens [14]. Proper inflammation response is beneficial for maintaining homeostasis, whereas excessive inflammation can exacerbate airway injury and promote systemic infection [15, 16]. Although systemic inflammation caused by SS2 has been studied, the molecular mechanism by which the airway epithelium coordinates airway inflammation elicited by SS2 remains unclear.

We recently identified that vimentin, the type III intermediate filament (IF) family protein, was necessary for SS2 to penetrate the murine airway epithelium [17]. The penetration of bacteria across the airway epithelium is attributed to the increased permeability of the epithelial barrier, and excessive inflammatory response is one of the important factors affecting the barrier’s permeability [18, 19]. As a cytoskeletal protein, vimentin is involved in maintaining cell morphology, the mechanical integrity of cells, and cell differentiation [20]. Numerous studies have demonstrated that vimentin also functions as a scaffold for signaling molecules. A previous study showed that vimentin binds phosphorylated ERK to prevent dephosphorylation [21]. The LRR domain of vimentin at the cell plasma membrane of colon epithelial cells interacts with nucleotide oligomerization domain protein 2 (NOD2) to regulate its activity and downstream NF-κB signaling [22]. However, the role of vimentin in SS2-induced airway epithelial inflammation has not been well characterized.

Our study found that SS2-infected vimentin knockout mice show less lung damage and neutrophils than wild type mice, and the levels of proinflammatory cytokines in vimentin knockout swine tracheal epithelial cells (VIM KO STEC) and the lungs of infected vimentin null mice were significantly reduced. In addition, proper vimentin localization is critical for proinflammatory cytokine and chemokine production. Finally, vimentin regulates the activation of NF-κB by increasing the transcription of *NOD2* to regulate the production of cytokines and chemokines, thereby promoting airway injury and systemic infection caused by SS2.

## Materials and methods

### Bacterial strains and cell lines

The *Streptococcus suis* serotype 2 (SS2) virulent strain ZY05719 was isolated from dead pigs during an outbreak of the streptococcal disease in Sichuan Province, China, in 2005. SS2 was grown in Todd-Hewitt Broth (THB, Becton Dickinson, Franklin Lakes, NJ, USA) to mid-log phase (an optical density at 600 nm (OD_600_) of 0.4–0.8) at 37 °C. Then, the bacteria were washed thrice with PBS and resuspended in DMEM unless otherwise indicated.

To obtain vimentin knockout swine tracheal epithelial cells (VIM KO STEC), STEC were infected with lentivirus containing recombinant lentiCRISPR plasmids and the single clone was detected by Western blot and immunofluorescence [17]. VIM KO STEC and STEC were cultured in high glucose Dulbecco modified Eagle medium (DMEM, Gibco, Grand Island, NY, USA) supplemented with 10% heat-inactivated fetal bovine serum (FBS, Gibco) in 5% CO2 at 37 °C. Cells were digested with trypsin for passage or experiments.

### Animal experiments

Four to six-week-old wild-type C57BL/6J (VIM^+/+^) and vimentin knockout (VIM^−/−^) mice were purchased from GemPharmatech (Nanjing, China). All mice were housed in the Barrier Environment at the Laboratory Animal Center of Nanjing Agricultural University. In a mouse intranasal infection model, SS2 can penetrate the tracheal epithelium and cause systemic infection at 24 h post-infection (hpi) [17, 23]. Based on this model, six VIM^+/+^ mice or six VIM^−/−^ mice were randomly separated into two groups, and the mice were intranasally inoculated with PBS or SS2 (1 × 10^9^ CFU) for 24 h.

### Histopathological analysis

For histopathological analysis, the lungs of mice were fixed and embedded in paraffin to create paraffin blocks, and the blocks were then sectioned and subjected to hematoxylin and eosin (H&E) staining or neutrophil immunohistochemistry.

### Analysis of bronchoalveolar lavage fluid (BALF)

For quantification of neutrophils in the BALF of mice, a 24 G needle bronchoalveolar catheter was used. The lavage fluid was obtained by inserting the catheter from the trachea and injecting 500 μL of sterile PBS. The process was repeated 3 times. The cell pellet in the lavage fluid was collected by centrifugation and treated with ammonium-chloride-potassium (ACK) red blood cell lysis buffer for 3 min at room temperature. After centrifugation, 1 × 10^6^ cells were incubated with FITC Anti-Mouse CD11b and APC Anti-Mouse Ly-6G (Gr-1) antibodies (Proteintech, Wuhan, China) at 4 °C for 1 h. After being washed with PBS, the cells were counted using a CytoFLEX flow cytometer (Beckman, Indianapolis, IN, USA). The data were analyzed by FlowJo V10 software (Tree Star Inc., San Carlos, CA, USA).

### RT-qPCR

The whole-lung homogenates of VIM^+/+^ mice and VIM^−/−^ mice, STEC, and VIM KO STEC infected with SS2 for 2 h were lysed with TRIzol (Vazyme Biotech Co., China) and total RNA was extracted according to the protocol provided by the manufacturer. HiScript Q RT SuperMix for qPCR (+ gDNA wiper) (Vazyme Biotech Co.) was used to synthesize cDNA. cDNA was amplified with ChamQ Universal SYBR qPCR Master Mix (Vazyme Biotech Co.) on a 7300 Real-Time PCR System (Applied Biosystems, Foster City, California, USA). The primer sequences used in qPCR are listed in Table 1. Expression was normalized to the expression of the *GAPDH* gene, and relative quantification compared to the uninfected cells was calculated using the 2^−ΔΔCt^ method [24].

**Table 1:** **Primers in this study**

Primer name	Sequence (5ʹ–3ʹ)
*Primers used for detection of transcription of genes in STEC*	
GAPDH F	TGGTCACCAGGGCTGCTT
GAPDH R	CATGTAGTGGAGGTCAATGAAGG
IL-8 F	ATTCCACACCTTTCCACCCC
IL-8 R	CCACTTTTCCTTGGGGTCCA
IL-6 F	AAATGTCGAGGCTGTGCAGA
IL-6 R	TCCACTCGTTCTGTGACTGC
TNF-α F	GCACTGAGAGCATGATCCGA
TNF-α R	GAAGGAGAAGAGGCTGAGGC
NOD2 F	CGTCTGCAAGGCTCTTTACTTG
NOD2 R	GCCGTCGGTCAATTTGTTGT
Vimentin F	CAGATCCAGGAACAGCACGT
Vimentin R	GAGAGGTCGGCAAACTTGGA
*Primers used for detection of transcription of mice genes*	
mGAPDH F	GGTGGAGCCAAAAGGGTCAT
mGAPDH R	GGGGGCTAAGCAGTTGGTG
mIL-6 F	TGGTCTTCTGGAGTACCATAGC
mIL-6 R	CTGTGACTCCAGCTTATCTCTTG
mKC F	ACTCAAGAATGGTCGCGAGG
mKC R	ACTTGGGGACACCTTTTAGCA
mNOD2 F	TCTGGAGGTTTGGCTTCGAG
mNOD2 R	ACAACAAGAGTCTGGCGTCC
mIL-1β F	ACTCAACTGTGAAATGCCACCT
mIL-1β R	TGTGCTGCTGCGAGATTTGA
mTNFα F	ACTGAACTTCGGGGTGATCG
mTNFα R	TGAGGGTCTGGGCCATAGAA

### Enzyme-linked immunosorbent assay (ELISA)

The concentrations of murine IL-6, TNF-α, and KC protein in the whole-lung homogenates of SS2-infected mice were determined using commercial ELISA kits (Fankewei, Shanghai, China) according to the manufacturer’s instructions. Assays were performed in triplicate for each independent experiment.

### The effect of SS2 on vimentin in the cell membrane of STEC

Confluent STEC in six-well plates were infected with SS2 (MOI 50:1), followed by incubation at 37 °C in 5% CO_2_ for the indicated time. The membrane proteins of STEC were isolated with the membrane and cytosolic protein extraction kit (KeyGEN Biotech, Nanjing, China). The extracted proteins were denatured then subjected to SDS-PAGE.

### Western blots

Differently treated STEC or VIM KO STEC were washed with cold PBS and lysed with RIPA (containing protease and phosphatase inhibitors) (KeyGEN Biotech, Nanjing, China) for 5 min on ice. RIPA lysis buffer (containing protease and phosphatase inhibitors) was added to 100 mg of lung tissue fragments to extract mouse lung tissue proteins. The protein concentration was determined using a BCA protein assay kit (Thermo Fisher, Waltham, MA, USA). After SDS-PAGE separation, the target protein bands on the polyacrylamide gel were cut and transferred to PVDF membranes (Millipore, Darmstadt, Germany) using the semi-dry transfer method. The membranes were blocked with 3% bovine serum albumin (BSA) in TBST for 2 h. Then, the membranes were incubated with appropriate primary antibodies at 4 °C overnight, followed by a corresponding secondary antibody at room temperature (RT) for 1 h. Immunoblotting was detected by ECL Femto-Detect™ Western Blotting Substrate (Engibody Biotechnology) and imaged on ChemiDoc Touch Imaging System (Bio-rad).

The information on the antibodies used is listed below: anti-vimentin rabbit polyclonal antibody (1:2000; Cat. No. 10366-1-AP) was purchased from Proteintech; NF-κB p65 (L8F6) mouse monoclonal antibody (mAb) (1:2000; Cat. No. 6956T) was purchased from Cell Signaling Technology (Beverly, MA, USA); phospho-NF-κB p65 (Ser536) rabbit polyclonal antibody (1:1000; Cat. No. TA2006) were purchased from Abmart (Shanghai, China); anti-GAPDH mouse mAb (1:5000; Cat. No. AT0002), anti-beta actin mouse mAb (1:5000; Cat. No. AT0001), HRP-conjugated goat anti-rabbit (1:5000; Cat. No. AT0097) and goat anti-mouse IgG antibody (1:5000; Cat. No. AT0098) were purchased from Engibody Biotechnology (Milwaukee, WI, USA).

### Immunofluorescence staining

STEC grown on coverslips were washed three times with PBS after different treatments. The cells were fixed in cold methanol for 20 min and blocked with 3% BSA for 2 h. Cells were incubated with anti-vimentin rabbit polyclonal antibody (1:400, Proteintech, Wuhan, China) at 4 °C overnight and then with DyLight 488-conjugated goat anti-rabbit IgG antibody (1:400, Abbkine, Wuhan, China) at RT for 1 h. The nuclei were stained with 4',6-Diamidino-2-phenylindole (DAPI, Beyotime, Nanjing, China). The coverslips were mounted using ProLong Gold antifade reagent (Invitrogen, Carlsbad, CA, USA) and imaged with a fluorescence microscope (Axio Observer 7; LD Plan-NEOFLUAR 40 × /0.6; ZEISS, Germany).

### Efficacy of WFA

STEC were pretreated with 2.5 μM or 5 μM WFA (MCE, China) for 1 h, then infected with SS2 at an MOI of 50:1 for 2 h. The immunofluorescence, RT-qPCR, or Western blot were performed to determine vimentin distribution, proinflammatory cytokines, chemokines transcription, or the activation of NF-κB.

### Statistical analysis

Statistical analysis was performed on GraphPad Prism 7 software (La Jolla, CA, USA). The data shown are presented as the mean of three independent experiments ± standard deviation (SD). Based on the normality test, the differences between the two groups were determined by an unpaired *t*-test. One-way ANOVA with Dunnett multiple comparison test or two-way ANOVA with Sidak multiple comparison test was used to determine the differences between more than two groups. Differences were considered significant when *P* < 0.05.

## Results

### Vimentin promotes SS2-induced lung inflammation and injury

**Figure 1:**
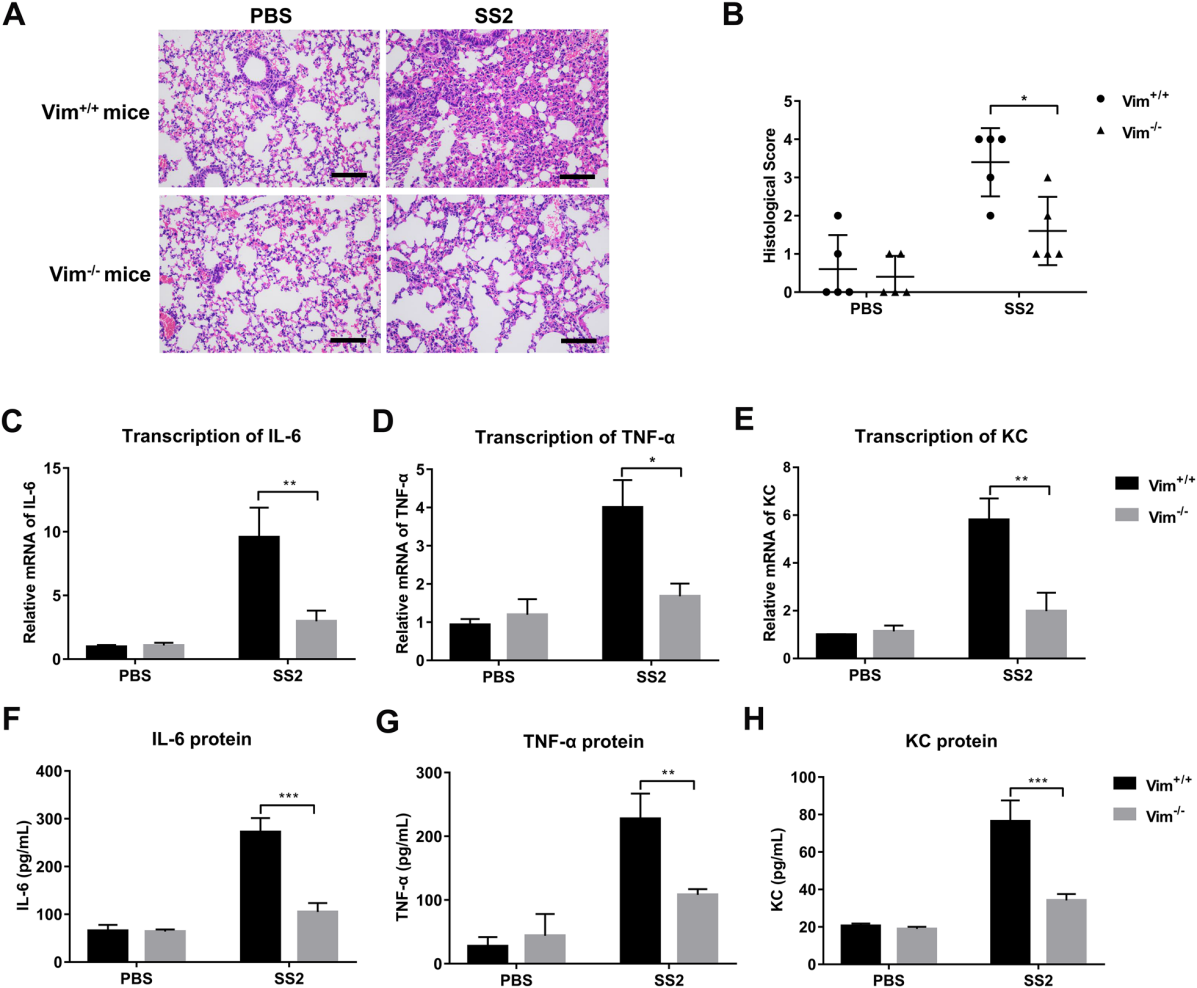
**Vimentin contributes to SS2-induced inflammation and injury in the airway of infected mice.** Vim^+/+^ mice and Vim^−/−^ mice (*n* = 3 mice/group) were intranasally challenged with PBS or SS2 for 24 h. **A** Hematoxylin and eosin (H&E) staining of lung sections of infected mice. Scale bar, 100 μm. **B** Blind scoring of the lung tissue section. Points represent the score of five random fields of lung tissue sections from 3 mice. Scored as follows: normal = 0, mild = 1, moderate = 2, severe = 3 and very severe = 4. Transcription of proinflammatory cytokines *IL-6* (**C**), *TNF-α* (**D**), and *KC* (**E**) in the lungs of infected mice were detected by RT-qPCR. The expression of proinflammatory cytokines IL-6 (**F**), TNF-α (**G**), and KC (**H**) in the lungs of infected mice were detected by ELISA. Data are representative or are presented as the mean ± SD. *, *P* < 0.05; **, *P* < 0.01; ***, *P* < 0.001.

To explore whether vimentin is required for the inflammatory response in the lung, wild-type C57/BL6J (Vim^+/+^) and vimentin knockout C57/BL6J (Vim^−/−^) mice were intranasally challenged with 1 × 10^9^ CFU SS2, and the lung inflammation and injury were observed by H&E staining. Histopathological examination shows that the lungs of infected Vim^+/+^ mice had more peribronchial accumulations of inflammatory cells and more severe alveolar damage characterized by interstitial edema, alveolar collapse, and hemorrhage than in Vim^−/−^ mice (Figures 1A and B).

We sought to determine whether Vim^−/−^ mice show diminished transcription of proinflammatory cytokines *IL-6*, *TNF-α*, and chemokine *KC* in lungs by RT-qPCR. As shown in Figures 1C–E, the transcript level of *interleukin-6* (*IL-6*), *tumor necrosis factor-alpha* (*TNF-α*), and *keratinocyte-derived chemokine* (*KC*, the IL-8 homolog) in the lungs of SS2-infected Vim^−/−^ mice were significantly lower than those in the lungs of Vim^+/+^ mice. Enzyme-linked immunosorbent assay (ELISA) shows significantly more IL-6, TNF-α, and KC protein in the whole-lung homogenates prepared from SS2-infected Vim^+/+^ mice (Figures 1F–H). Collectively, these results demonstrate that loss of vimentin prevents lung injury and inflammation in mice.

### Vimentin deficiency suppresses the recruitment of neutrophils in airway epithelium

**Figure 2:**
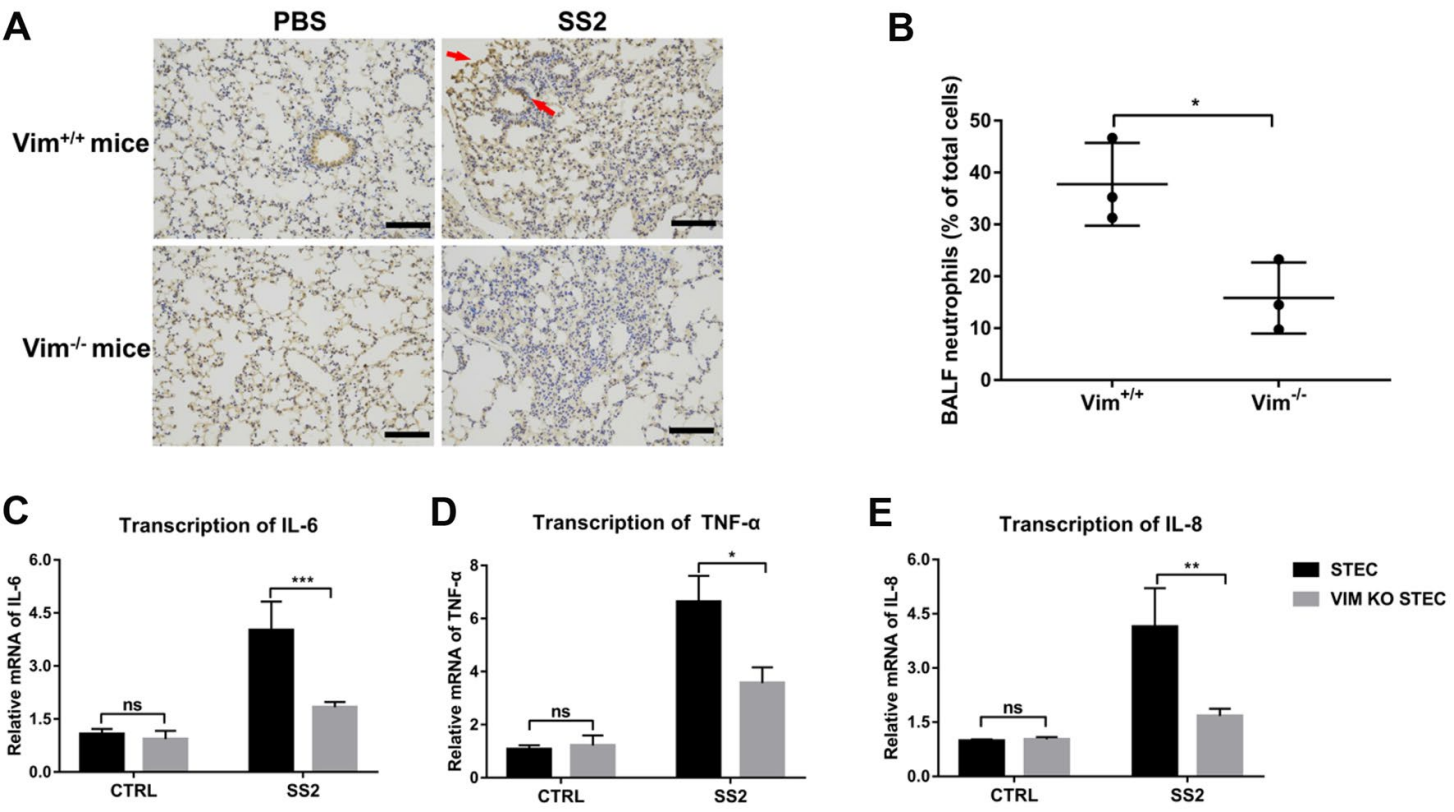
**The deficiency of vimentin prevents the recruitment of neutrophils in the airway.** **A** Immunohistochemistry of the lung of PBS- or SS2-infected mice (*n* = 3 mice/group) at 24 hpi using the neutrophil marker Ly-6G. Red arrows indicate the neutrophils. Scale bar, 100 μm. **B** Neutrophil numbers in BALF of infected mice were quantified by FACS. Anti-Mouse CD11b and APC Anti-Mouse Ly-6G (Gr-1) antibodies were used to label neutrophils. **C**–**E** RT-qPCR detected the transcription of proinflammatory cytokines *IL-6* (**C**), *TNF-α* (**D**), and *IL-8* (**E**) in STEC and VIM KO STEC infected with SS2 (MOI 50) for 2 h. Data are representative or are presented as the mean ± SD. ns, not significant; *, *P* < 0.05; **, *P* < 0.01; ***, *P* < 0.001.

Neutrophils have been long thought to play a predominant role in the inflammatory response caused by SS2 infection [25, 26]. We wondered whether there was a difference in the number of neutrophils recruited in the lungs of SS2-infected Vim^+/+^ and Vim^−/−^ mice. Immunohistochemistry of the lung using the neutrophil marker Ly-6G after infection with SS2 shows a massive accumulation of neutrophils in the lungs of Vim^+/+^ mice, while relatively few neutrophils were recruited to the lungs of Vim^−/−^ mice (Figure 2A). To further quantify the neutrophil recruitment into the lungs, we performed a systematic flow cytometric analysis of bronchoalveolar lavage fluid (BALF). Vim^+/+^ mice challenged with SS2 had significantly increased neutrophils in BALF than SS2-challenged Vim^−/−^ mice (Figure 2B).

To explore whether vimentin plays a role in cytokine production by tracheal epithelial cells, VIM KO STEC and STEC were infected with SS2 for 2 h, and the transcript levels of *IL-6*, *TNF-α*, and *IL-8* were assessed by RT-qPCR. Transcript levels of *IL-6*, *TNF-*α, and *IL-8* in VIM KO STEC were significantly lower than those in STEC (Figures 2C–E); however, transcription of *IL-6*, *TNF-α*, and *IL-8* was still induced in VIM KO STEC, which suggests that vimentin is one factor involved in inflammation in the airway. These data suggest that the vimentin of epithelial cells promotes the production of cytokines.

### Vimentin reassembles in swine tracheal epithelial cells in response to SS2 infection

The expression of vimentin in STEC infected with SS2 was determined by Western blot and RT-qPCR. The results show that the total protein level of vimentin and the transcription of the *vimentin* gene did not change at all time points during infection (1–4 hpi) (Additional files 1, 2).

**Figure 3:**
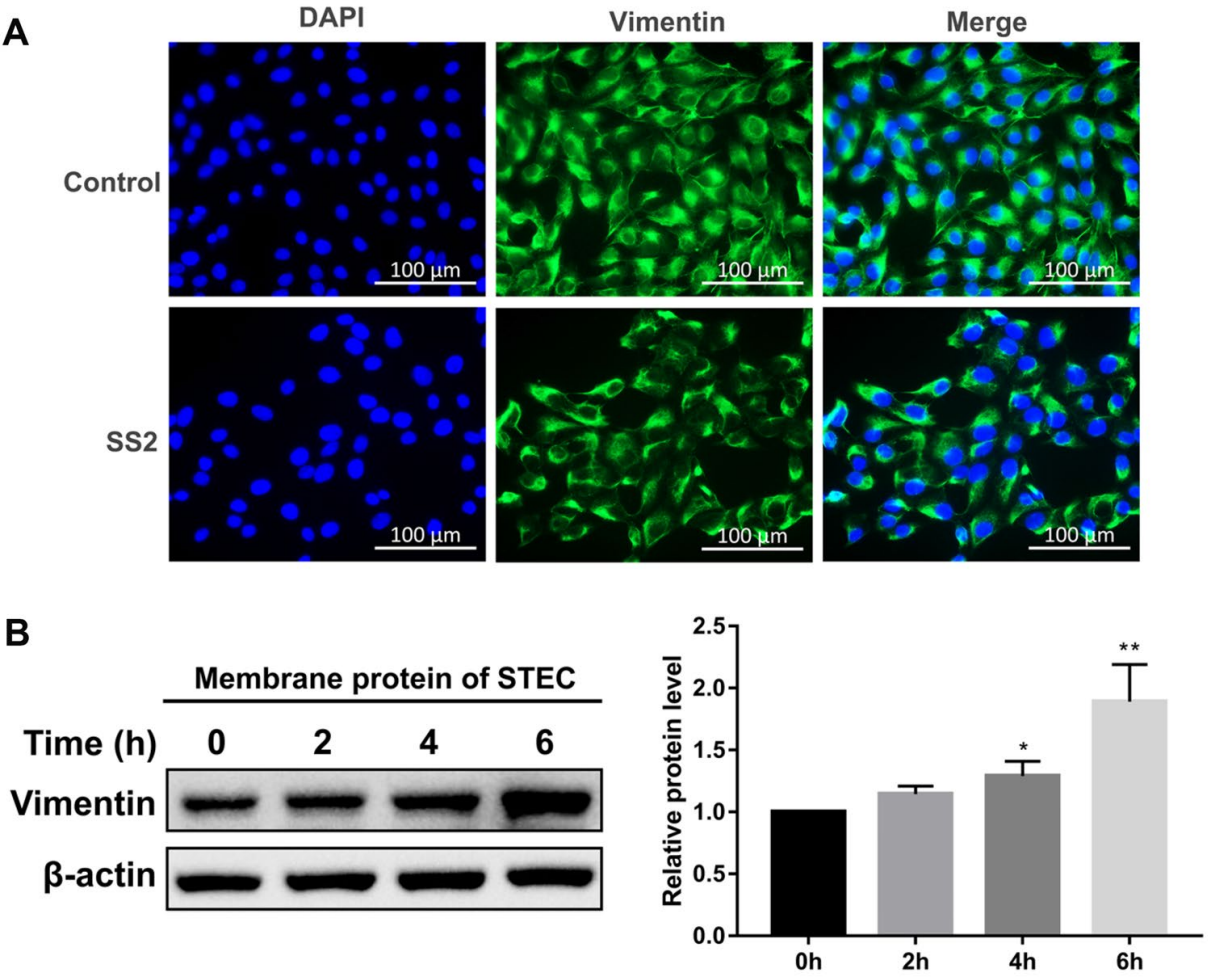
**Vimentin reassembles at the cell membrane of swine tracheal epithelial cells in response to SS2 infection.** **A** The distribution of vimentin (green) in STEC was detected by immunofluorescence staining at 2 hpi. The cell nuclei were stained with DAPI (blue). Scale bar, 100 μm. **B** STEC were infected with SS2 (MOI 50) for the indicated time. The expression of vimentin in the cell membrane of STEC was detected by Western blot. Band intensities relative to the uninfected group were analyzed. Data are representative or are presented as the mean ± SD. *, *P* < 0.05; **, *P* < 0.01.

The distribution of vimentin is involved in the infection of various pathogenic bacteria [27–29]. To visualize the localization of vimentin in tracheal epithelial cells, we performed immunofluorescence staining of uninfected and SS2-infected STEC. We observed that vimentin protein was present throughout the cytoplasm of uninfected STEC, whereas, in the SS2-infected STEC, the vimentin lost its uniform intermediate fiber structure and clustered on one side of the cell membrane (Figure 3A). To characterize the distribution of vimentin in the cell membrane, we analyzed the time-dependent expression of vimentin in the cell membrane of SS2-infected STEC. As shown in Figure 3B, SS2 infection significantly increased vimentin expression at the membrane of STEC compared to the uninfected control. These results indicate that SS2 induces vimentin to cluster on the cell membrane of STEC.

### Proper localization of vimentin in STEC is required for the transcription of *IL-6*, *TNF-α*, and *IL-8*

**Figure 4:**
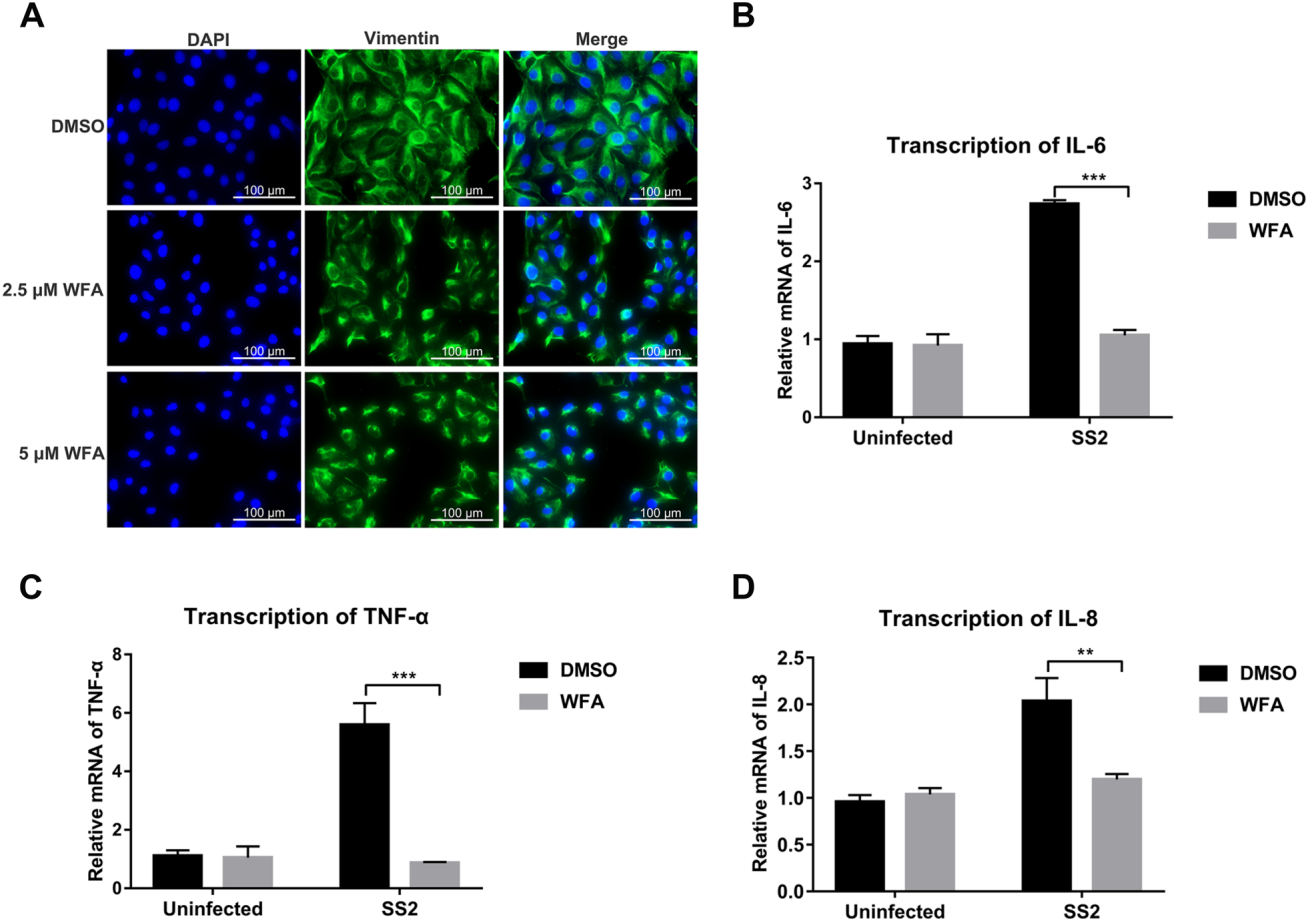
**Disruption of vimentin localization inhibits the transcription of cytokines in STEC.** **A** The effect of vimentin inhibitor WFA on the distribution of vimentin (green) in uninfected STEC was examined by immunofluorescence. The cell nuclei were stained with DAPI (blue). Scale bar, 100 μm. STEC pretreated with DMSO or 2.5 μM WFA for 1 h were infected with SS2 (MOI 50) for 2 h, and the transcription of *IL-6* (**B**), *TNF-α* (**C**), and *IL-8* (**D**) was determined using RT-qPCR. The same volume of DMSO treatment is the control. Data are representative or are presented as the mean ± SD. **, *P* < 0.01; ***, *P* < 0.001.

Next, we explored whether vimentin reassembly is required to produce inflammatory cytokines or chemokines induced by SS2 in STEC. We used the vimentin inhibitor, withaferin A (WFA), to perturb the assembly of the vimentin intermediate filament. WFA is a natural steroidal lactone that can covalently bind to the conserved α-helical coil 2B domain of vimentin to inhibit its assembly [30]. Uninfected STEC were treated with different concentrations of WFA to explore the concentration of WFA required to destroy vimentin in STEC. As shown in Figure 4A, 2.5 μM WFA was sufficient to lead the uniform cytoplasmic distribution of the vimentin network in control cells to present perinuclear distribution, and 5 μM WFA caused a complete collapse of the vimentin filament. Next, we examined the effect of 2.5 μM WFA on the transcriptional level of *IL-6*, *TNF-α*, and *IL-8* in SS2-infected STEC. WFA pretreatment could significantly decrease the transcription of *IL-6*, *TNF-α*, and *IL-8* in STEC after SS2 infection compared with that in DMSO-pretreated cells (Figures 4B–D). Altogether, these observations suggest that the proper vimentin localization is essential for SS2-induced cytokine production in STEC.

### Reassembly of vimentin contributes to the transcription of *NOD2* and phosphorylation of NF-κB protein

**Figure 5:**
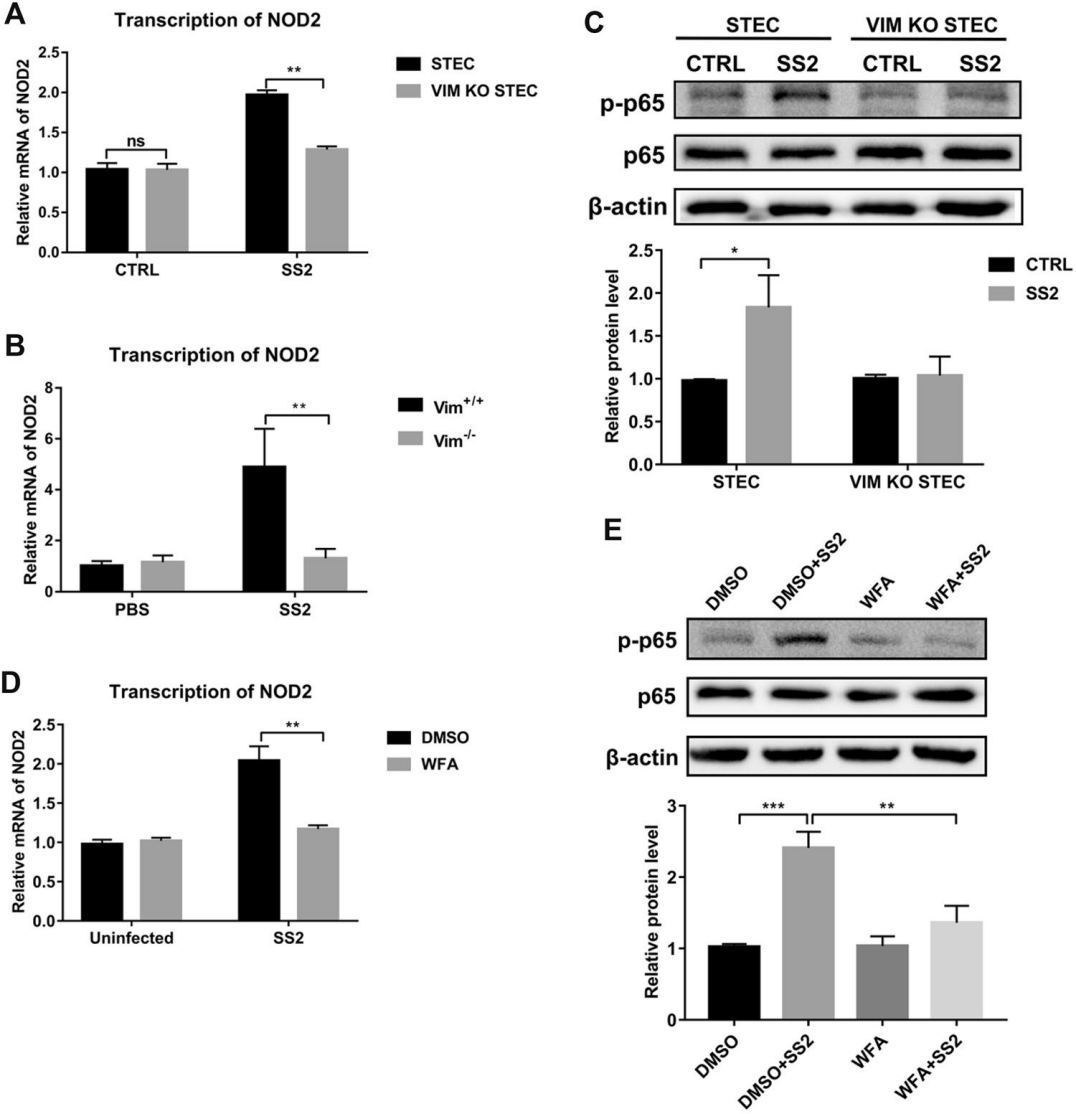
**Reassembly of vimentin contributes to the transcription of *NOD2* and phosphorylation of p65.** **A** STEC and VIM KO STEC were infected with SS2 (MOI 50) for 2 h, and the transcription of *NOD2* was detected by RT-qPCR. **B** Transcription of *NOD2* in the lungs of PBS- or SS2-infected mice was detected by RT-qPCR. **C** STEC and VIM KO STEC were infected with SS2 (MOI 50) for 2 h, and the expression of phospho-p65 and p65 was analyzed by Western blot. The graph indicated the relative protein level of phospho-p65. **D**, **E** STEC pretreated with DMSO or 2.5 μM WFA for 1 h were infected with SS2 (MOI 50) for 2 h, the transcription of *NOD2* was determined using RT-qPCR (**D**), and the expression of phospho-p65 and p65 was analyzed by Western blot (**E**). The graph indicated the relative protein level of phospho-p65 (**E**). Data are representative or are presented as the mean ± SD. *, *P* < 0.05; **, *P* < 0.01; ***, *P* < 0.001.

Studies have shown that vimentin interacts with NOD2 [22], and NOD2 activates nuclear factor NF-κB, leading to inflammation-related diseases [31]. We determined the effect of vimentin on the transcription of *NOD2* in SS2-infected STEC and murine lungs. As shown in Figure 5A, the *NOD2* transcript levels were significantly increased in SS2-infected STEC compared with VIM KO STEC. Similarly, the *NOD2* transcript level in SS2-infected Vim^+/+^ mice was significantly higher than in SS2-infected Vim^−/−^ mice (Figure 5B). These experiments indicate that vimentin is an important factor affecting NOD2 transcription.

To further elucidate vimentin’s role in activating NF-κB, we compared the effect of SS2 infection on the phosphorylation level of NF-κB protein p65 in STEC and VIM KO STEC. We observed that SS2-infected STEC had increased levels of phosphorylated p65 protein (Figure 5C). Next, we found a failure increase of *NOD2* transcription and phosphorylated p65 protein in STEC after destroying the vimentin filaments using 2.5 μM WFA (Figures 5D, E). Together, these results demonstrate that vimentin is involved in activating the NOD2/NF-κB pathway in the airway epithelium.

## Discussion

SS2 can cause systemic infection through the swine respiratory tract [17]. Excessive inflammation and increased permeability of the airway epithelial barrier are the major pathophysiological events during SS2 infection [32]. Given that the ability of SS2 to penetrate the tracheal epithelium of vimentin null mice and enter various tissues was markedly reduced [17], we hypothesize that vimentin is involved in SS2-induced excessive inflammatory response. Here, we found that vimentin contributed to lung damage, the number of neutrophils and expression of proinflammatory cytokines and chemokines in the lungs of mice and STEC after SS2 infection. Our further studies revealed a unique requirement for proper distribution of vimentin to produce neutrophil chemokine in the airway epithelium. Vimentin knockout and pharmacological disruption of vimentin filaments in STEC failed to increase the expression of NOD2 and phosphorylated p65 protein. All these results corroborate that vimentin plays a central role in the SS2-induced airway inflammation and systemic infection through NOD2/NF-κB signaling.

Our recent studies found that vimentin prompts bacterial penetration of the tracheal epithelium by interacting with autolysin of SS2 [17]. Here, we further demonstrate that vimentin facilitates the expression of neutrophil chemokine and lung damage in SS2-infected airway epithelium. Cytokines produced by airway epithelium and immune cells are responsible for recruiting neutrophils to the infection site [35]. We propose that vimentin in the airway epithelium is correlated with the recruitment of immune cells. This is supported by the fact that vimentin was involved in the transcription of the cytokines *IL-6*, *TNF-α*, and chemokine *IL-8* in STEC. In a murine infection model, the reduction of pulmonary neutrophils may be partially related to the decreased production of airway epithelial chemokines. Vimentin is also reported to regulate cell migration [36, 37]. Another possible mechanism for the reduction of neutrophils in the lung of vimentin null mice may be a lower migration capacity of the neutrophils. Further investigation will be required to define the exact contribution of these mechanisms involved.

Since vimentin null mice exhibited less lung pathological damage, we speculate that failure to produce cytokines caused this protection. Vimentin participated in the transcription and production of the proinflammatory cytokines IL-6, TNF-α, and chemokine KC in the lungs of SS2-infected mice. Recently, the dynamic role of vimentin has also been increasingly confirmed. In addition to maintaining cell morphology as a cytoskeletal protein, the immune function of vimentin is increasingly being demonstrated. Vimentin interacts with components of the NLRP3 inflammasome and is required for inflammasome activation in macrophage cells [38]. Phosphorylated vimentin activates TGF-β signaling, leading to metastasis and PDL1 expression for immune suppression in lung adenocarcinoma [39]. Extracellular vimentin secreted by activated human macrophages facilitated the killing of bacteria and the generation of oxidative metabolites [40]. Considering the fact that the excessive inflammatory response leads to a leaky barrier [18, 41], the role of vimentin in airway inflammation caused by SS2 may cooperate with its role as the receptor of autolysin to increase the permeability of the airway epithelium, thereby facilitating the systemic infection caused by SS2.

In airways, tissue resident immune cells such as macrophages and dendritic cells (DC) defend against airborne pathogens and produce proinflammatory cytokines that in turn recruit dendritic cells, monocyte derived macrophages and neutrophils [42]. While those professional immune cells play a major role in the inflammatory response, it has become evident that the airway epithelial cells are also actively involved in coordinating both innate and adaptive immune response by releasing various inflammatory mediators and cytokine/growth factors [43, 44]. Consistent with the murine infection model results, vimentin in STEC contributed to the transcription of *IL-6*, *TNF-α*, and *IL-8*. Reduced production of epithelial cytokines may be responsible for poor neutrophil recruitment in the airway of Vim^−/−^ mice which leads to protection from lung inflammation and injury. Notably, the transcription of proinflammatory cytokines was still increased in VIM KO STEC compared to uninfected cells, implying that vimentin is only one contributing factor affecting proinflammatory cytokines in the airway epithelium.

As a major cytoskeletal protein, vimentin has historically been viewed as a cytoplasmic protein that regulates cell mechanics, motility, and intracellular signaling [45]. Growing evidence indicates that cell surface vimentin is implicated in host–pathogen interactions, thereby promoting the adhesion, entry, or transport of viruses or pathogenic bacteria such as SARS-CoV-2, *Listeria monocytogenes*, and meningitic *Escherichia coli* K1 [27, 29, 46]. Intriguingly, we found that vimentin clustered at the cell membrane of SS2-infected STEC. The cluster of vimentin was related to the production of proinflammatory cytokines in the airway epithelium, which was similar to the role of vimentin in the production of chemokine in brain endothelial cells [47].

The underlying mechanisms by which respiratory pathogens elicit airway epithelial inflammation appear different [13, 48–50]. In this study, vimentin promoted the transcription of *NOD2* in STEC and the lungs of mice. It has been reported that vimentin binds to NOD2 [22], and NOD2 is required for subsequent RIP2-dependent activation of NF-κB signaling, a classic inflammation-related pathway [51–53]. We demonstrate that vimentin is involved in the phosphorylation of NF-κB protein p65, as deletion of vimentin or disruption of vimentin filaments in STEC fails to activate NF-κB signaling. It has been shown that vimentin regulates lung inflammation by regulating the NLRP3 inflammasome [38] and acts as the scaffolds of various signaling molecules to regulate signaling pathways [21, 30]. The precise contribution of vimentin to airway inflammation would be a topic for future investigation. Our results provide insights into the molecular mechanism by which vimentin promotes the excessive inflammation and lung injury via NOD2/NF-κB signaling during SS2 infection, which was further supported by the study that vimentin in human cerebral microvascular endothelial cells contributes to the pathogenesis of meningitis caused by group B *streptococcus* (GBS) (38). Excessive airway inflammation and subsequent systemic infections caused by various bacteria and viruses account for extensive mortality and morbidity worldwide, seriously threatening public health [54, 55]. Our studies reveal that vimentin may play the same role in airway inflammation caused by other agents and provide the theoretical groundwork for exploiting vimentin as a potential therapeutic target for airway infection.

## Supplementary Information

**Additional file 1.** **The transcription of *vimentin* in STEC infected with SS2.** Data are representative or are presented as the mean ± SD. ns: not significant.

**Additional file 2. The protein quantification of vimentin in whole-cell extracts of STEC.** Data are representative or are presented as the mean ± SD. ns: not significant.

## Data Availability

All data generated or analysed during this study are included in this published article (and its additional information files).

## Abbreviations

*S. suis*: *Streptococcus suis*
SS2: *Streptococcus suis* Serotype 2
STSLS: Streptococcal toxic shock-like syndrome
IL-6: interleukin-6
TNF-α: tumor necrosis factor-alpha
KC: keratinocyte-derived chemokine
STEC: swine tracheal epithelial cells
VIM KO STEC: vimentin knockout swine tracheal epithelial cells
NOD2: nucleotide oligomerization domain protein 2
IF: intermediate filament
THB: Todd-Hewitt Broth
OD_600_: optical density at 600 nm
DMEM: Dulbecco modified Eagle medium
VIM^+/+^ mice: wild-type C57BL/6J mice
VIM^−^^/^^−^ mice: vimentin knockout mice
H&E: hematoxylin and eosin
BALF: bronchoalveolar lavage fluid
ELISA: enzyme-linked immunosorbent assay
BSA: bovine serum albumin
RT: room temperature
DAPI: 4',6-Diamidino-2-phenylindole
SD: standard deviation
