# Supplementary figures and images for: Vimentin affects inflammation and neutrophil recruitment in airway epithelium during Streptococcus suis serotype 2 infection
Source: Vet Res. 2023 Jan 30;54:7. doi: 10.1186/s13567-023-01135-3 (PMC9885403; doi:10.1186/s13567-023-01135-3)

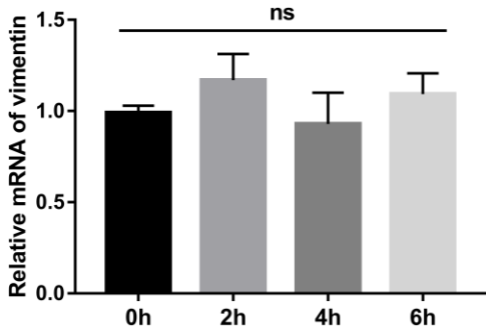

Supplement: Supplementary file 1 — Additional file 1. The transcription of vimentin in STEC infected with SS2. Data are representative or are presented as the mean ± SD. ns: not significant. [file 13567_2023_1135_MOESM1_ESM.pdf]

Total protein of STEC

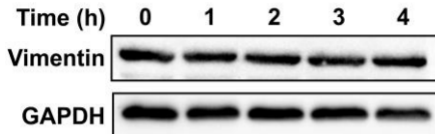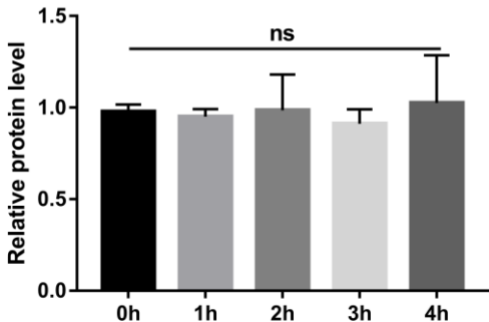

Supplement: Supplementary file 2 — Additional file 2. The protein quantification of vimentin in whole-cell extracts of STEC. Data are representative or are presented as the mean ± SD. ns: not significant. [file 13567_2023_1135_MOESM2_ESM.pdf]
